# Genome-wide association studies targeting the yield of extraembryonic fluid and production traits in Russian White chickens

**DOI:** 10.1186/s12864-019-5605-5

**Published:** 2019-04-04

**Authors:** Andrei A. Kudinov, Natalia V. Dementieva, Olga V. Mitrofanova, Olga I. Stanishevskaya, Elena S. Fedorova, Tatiana A. Larkina, Arina I. Mishina, Kirill V. Plemyashov, Darren K. Griffin, Michael N. Romanov

**Affiliations:** 1 Russian Research Institute of Farm Animal Genetics and Breeding Branch of the L. K. Ernst Federal Science Centre for Animal Husbandry, Pushkin, St Petersburg, 196601 Russia; 2 University of Helsinki, FI-00014 Helsinki, Finland; 3 School of Biosciences, University of Kent, Canterbury, Kent CT2 7NJ UK

**Keywords:** GWAS, Russian white chicken breed, Poultry, Day-old chick down colour, Yield of extraembryonic fluid, SNP

## Abstract

**Background:**

The Russian White is a gene pool breed, registered in 1953 after crossing White Leghorns with local populations and, for 50 years, selected for cold tolerance and high egg production (EL). The breed has great potential in meeting demands of local food producers, commercial farmers and biotechnology sector of specific pathogen-free (SPF) eggs, the former valuing the breed for its egg weight (EW), EL, age at first egg (AFE), body weight (BW), and the latter for its yield of extraembryonic fluid (YEF) in 12.5-day embryos, ratio of extraembryonic fluid to egg weight, and embryo mass. Moreover, its cold tolerance has been presumably associated with day-old chick down colour (DOCDC) – white rather than yellow, the genetic basis of these traits being however poorly understood.

**Results:**

We undertook genome-wide association studies (GWASs) for eight performance traits using single nucleotide polymorphism (SNP) genotyping of 146 birds and an Illumina 60KBeadChip. Several suggestive associations (*p* < 5.16*10^− 5^) were found for YEF, AFE, BW and EW. Moreover, on chromosome 2, an association with the white DOCDC was found where there is an linkage disequilibrium block of SNPs including genes that are responsible not for colour, but for immune resistance.

**Conclusions:**

The obtained GWAS data can be used to explore the genetics of immunity and carry out selection for increasing YEF for SPF eggs production.

**Electronic supplementary material:**

The online version of this article (10.1186/s12864-019-5605-5) contains supplementary material, which is available to authorized users.

## Background

Programmes for the conservation of genetic resources including local, rare and endangered breeds are gaining increasing importance for the preservation of biodiversity, especially in farm animals (e. g., [1, 2]). Such gene pool chicken breeds are characterised by variations in phenotypic diversity and, in comparison to commercial poultry, are distinguished by high viability, special quality of meat and eggs, and/or having unique genetic features and appearance [1, 3–6]. One such breed is the Russian White (RW), whose development began around 1929 from crossing local chickens with parental stocks of the White Leghorn breed imported from Denmark, the UK and USA [7] in the Pyatigorsk and Kuchino breeding centres. Before RW was approved as a genuine breed in 1953, more attention was paid in Pyatigorsk to increase egg production (EL), and in Kuchino to increase body weight (BW).

The RW population maintained at the Russian Research Institute of Farm Animal Genetics and Breeding (RRIFAGB) was put under strong selection pressure for 50 years (1953 to 2003) [3, 7, 8]. Derived from one founder, its breeding was carried out selecting for tolerance to cold in chicks [7]. Initially, the day-old chick down colour (DOCDC) was yellow but selection for cold tolerance led to individuals with white down [3], the trait being supposedly controlled by the recessive gene *sw* for snow-white down in the chick [9]. It is currently used to develop poultry lines for the production of viral vaccines in embryonated eggs. The use of developing chick embryos makes it possible to increase the vaccine production and to expand the spectrum of viruses cultivated in the laboratory.

Despite the importance of this breed for all the above reasons, there is little or no information on the factors affecting (a) the yield of allanto-amniotic fluid that serves as a raw material for the vaccine production, and (b) as a consequence, the viral antibody titre in this fluid [10–12]. To carry out effective selection for increasing the yield of extraembryonic fluid (YEF), the genetic factors affecting this parameter must be established. Furthermore, in breeding egg laying hens, there are certain economically relevant traits at extreme ends of the phenotypic spectrum, knowledge of the genetics of which could be of use to breeders. These include AFE, egg weight (EW) and BW, and to take these traits into account is determinative both in the production of biopreparations and in the use of eggs for food purposes.

Recently, with the development and application of high-throughput genotyping technologies, it has become possible to identify associations of genomic regions and loci with selected traits. Traditional approaches, such as microsatellites randomly distributed throughout the genome, previously found quantitative trait loci (QTLs) for many economically important traits [13–20]. Locating single nucleotide polymorphisms (SNPs) in functional and positional candidate genes allowed testing for associations with the trait itself [21–30]. With the advent of whole genome next generation sequencing, however, either through the intermediate of a SNP chip, or more recently (as technology has become cheaper) by sequencing individuals directly, genome-wide association studies (GWASs) are discovering new loci associated with specific traits [31–34].

In particular, SNP genotyping using the Illumina Chicken 60 K SNP iSelect BeadChip (Illumina, USA), along with whole genomic sequencing, has found associations with traits related to poultry immunity and phenotypic characteristics [35, 36]. Given the importance of gene pool lines in general and the ability to refine pre-existing genotype × phenotype associations by GWAS, such studies are essential. Taking into account the pivotal importance of the RW line specifically for the vaccine and foodstuff production as well as understanding the genetic basis of cold tolerance, GWAS using RW seems to be a priority. With this in mind, the aim of this study was to establish hitherto undiscovered associations in the genome of Russian gene pool chickens with the following traits: EW, YEF, ratio of extraembryonic fluid to egg weight (REFEW), embryo mass (EM), EL, AFE, BW and, because of its association with cold tolerance, DOCDC.

## Results

### Day-old chick down colour

**Fig. 1:**
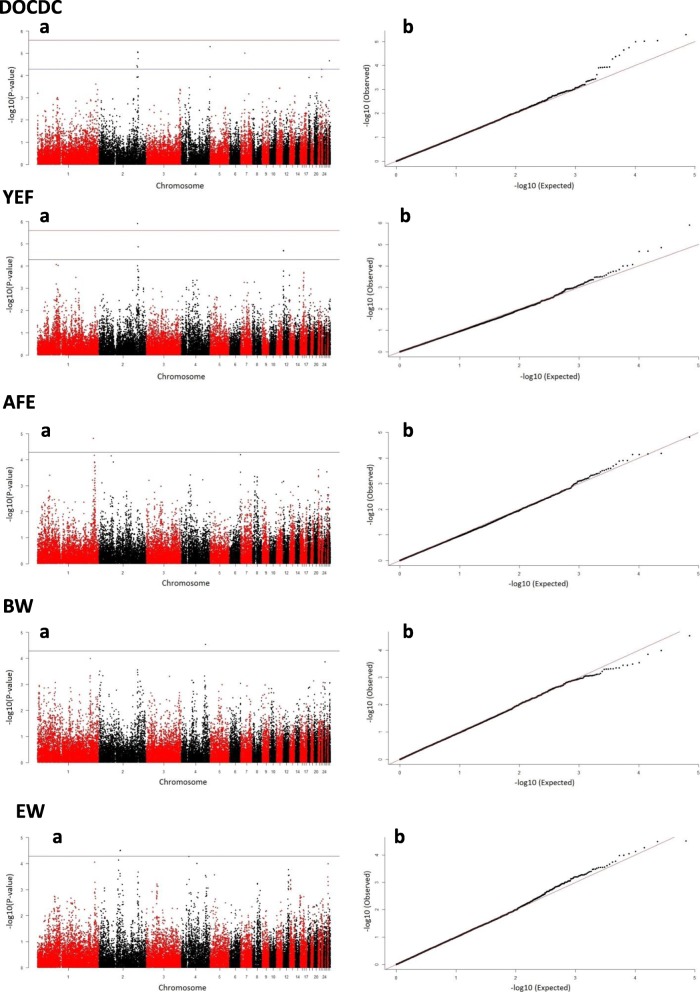
Manhattan (**a**) and Q-Q (**b**) plots of GWAS for DOCDC, YEF, AFE, BW and EW. Each dot on Manhattan plot represents a SNP according to chromosome. The horizontal red and blue lines are present significant (2.58*10^− 6^) and suggestive (5.16*10^− 5^) genome-wide associati*o*n thresholds, respectively

As a result of the genotyping of 146 RW females from 12 sires (Table 1) using 35,390 SNPs spread across 28 autosomes (Additional file 1: Table S1), five markers located on the chicken chromosome (GGA) 2 region were detected to be suggestively associated (*p* ≤ 4.2e-5) with DOCDC. The phenotypic variance explained by the set of SNPs was 11% (SE = 0.12). Three SNPs on GGA4 (rs16455118), GGA7 (rs317256404) and GGA28 (rs16209462) were also above suggestive line, with the phenotypic variance explained by them being 33% (SE = 0.18). The Manhattan and quantile-quantile (Q-Q) plots for DOCDC are presented in Fig. 1, and detailed information about the appropriate associated markers is given in Table 2. The observed genomic inflation factor (*λ*_g_) was 1.019.

**Table 1:** Number of half and full sibs animals with records within sire

Sire	No. of progeny	Half sibs	Groups of full sibs	Mean no. of animals within group	SD^a^
1	9	3	2	3.00	1.41
2	7	2	2	2.50	0.71
3	17	2	5	3.00	0.71
4	16	2	4	3.50	0.58
5	11	2	3	3.00	1.73
6	7	2	3	2.00	0.00
7	13	2	4	2.75	0.96
8	12	2	4	2.50	1.00
9	13	3	3	3.33	2.30
10	9	2	3	2.33	0.58
11	13	1	5	2.40	0.55
12	11	3	2	4.00	0.00
Total	138	+ 8 animals with unknown pedigree			

Table shows the number of half and full sibs in the analysed population. All 138 birds can be divided into 12 groups according to sire ancestry; within each sire several animals were from the same dam. Thus, groups of full sibs are groups of animals with the same sire and dam. Origin of 8 birds was not defined clearly and was set as unknown in the study

^a^*SD* standard deviation

**Table 2:** Significant and suggestive SNPs identified for traits in the RW chickens

Trait^a^	SNP	GGA^b^	Position (bp)	Alleles	MAF^c^	*p*-value	Candidate / nearest genes
DOCDC	rs314384321	2	123,194,966	C/T	0.05	8.97e-06^d^	*SLC7A13*
	rs15150566	2	122,907,241	A/G	0.05	9.57e-06^d^	*RALYL*
	rs15151359	2	124,071,457	A/G	0.05	1.78e-05^d^	*MMP16*
	rs16116752	2	119,793,622	A/G	0.06	3.76e-05^d^	*ZFHX4*
	rs14243963	2	122,016,783	A/G	0.07	4.26e-05^d^	*IMPA1*
	rs16455118	4	91,252,525	C/A	0.39	5.13e-06^d^	*DYSF*
	rs317256404	7	12,113,970	A/G	0.46	1.01e-05^d^	*PLEKHM3*
	rs16209462	28	556,300	A/G	0.01	2.20e-05^d^	*SPPL2B*
YEF	rs13730111	2	121,679,302	C/T	0.20	1.24e-06^e^	*ZNF704*
	rs316856766	2	123,425,663	A/G	0.18	1.41e-05^d^	*CA2*
	rs15630281	12	1,302,350	C/T	0.33	2.08e-05^d^	*PRKCD*
	rs315166929	12	1,006,316	C/T	0.46	2.14e-05^d^	*LOC107054345*
AFE	rs317931060	1	178,102,549	A/C	0.34	1.52e-05^d^	*FGF9*
BW	rs15619223	4	76,404,421	A/C	0.42	2.90e-05^d^	*LCORL*
EW	rs14201361	2	68,512,817	A/G	0.19	3.10e-05^d^	*KIAA1468*
	rs14200974	2	68,304,144	A/G	0.26	3.21e-05^d^	*PHLPP1*
	rs14439117	4	24,250,433	C/T	0.22	5.42e-05^d^	*TLL1*

^a^Traits studied: *DOCDC* day-old chick down colour, *YEF* yield of extraembryonic fluid, *AFE* age at first egg, *BW* body weight, *EW* egg weight

^b^Chicken chromosome

^c^Minor allele frequency

^d^Suggestive SNPs

^e^Significant SNP

**Fig. 2:**
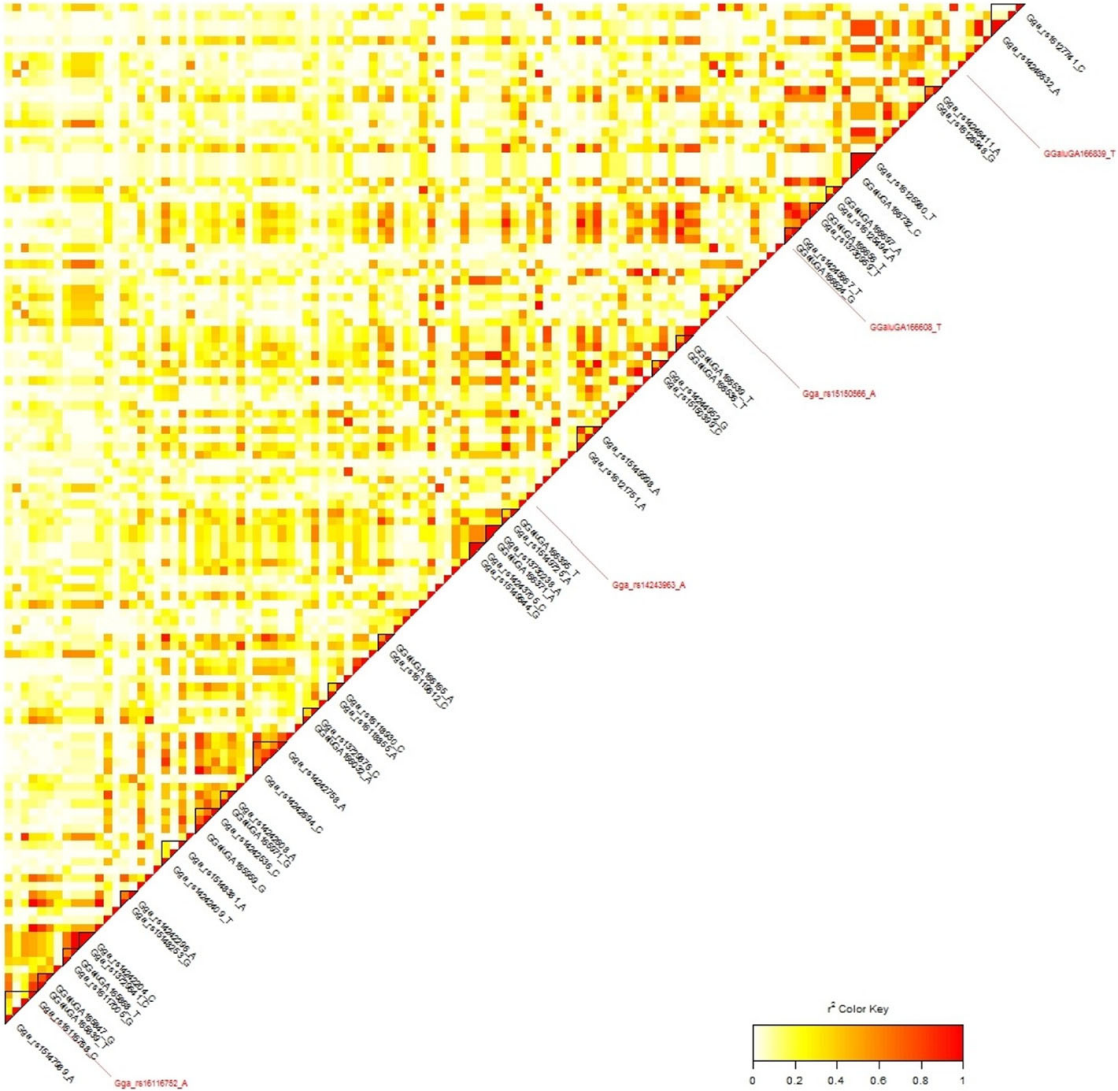
LD (r^2^) plot of markers on GGA2 associated with DOCDC. Haplotype blocks are shown as triangles with red sides. Colour inside of the triangle depends on level of r^2^ (LD). r^2^ closer to 1 has more red colour

The level of linkage disequilibrium (LD) on GGA2 was calculated for the region span ranging between 119.6 to 124.4 Mb (Fig. 2). The region contained 123 SNPs, five of which were suggestively associated with DOCDC. Only two LD blocks from 24 detected ones were selected for the further detailed analysis. The first 11.8-kb haplotype block bordered on the most suggestive SNP (rs314384321), and included the genes *LRRCC1* (leucine rich repeat and coiled-coil centrosomal protein 1) and *SLC7A13* (solute carrier family 7 member 13). The second 180.3-kb block, with the suggestive SNP rs16116752 inside, contained the *ZFHX4* (zinc finger homeobox 4) gene as well as two uncategorised non-coding RNA (ncRNA) loci, *LOC107052753* and *LOC101749450*. One suggestive SNP each was also found in the genes *DYSF* (dysferlin) on GGA4 (rs16455118), *PLEKHM3* (pleckstrin homology domain containing M3) on GGA7 (rs317256404) and *SPPL2B* (signal peptide peptidase like 2B) on GGA28 (rs16209462).

### Yield of extraembryonic fluid

Two regions locating on GGA2 between 121.4 and 121.8 Mb and on GGA12 between 0.9 and 1.2 Mb were detected to be associated with YEF (Table 2). On GGA2, the SNP rs13730111 passed the significant test (*p* = 1.24e-6), while rs316856766 passed the suggestive line (*p* = 1.4e-5). Estimated explained phenotypic variance for both markers was 17.9% (SE = 0.20). Suggestive association on GGA12 was detected for two markers, rs15630281 and rs315166929, with explained phenotypic variance being 10.6% (SE = 0.12). The respective Manhattan and Q-Q plots are shown in Fig. 1, with the observed *λ*_g_ being equal to 0.93.

**Fig. 3:**
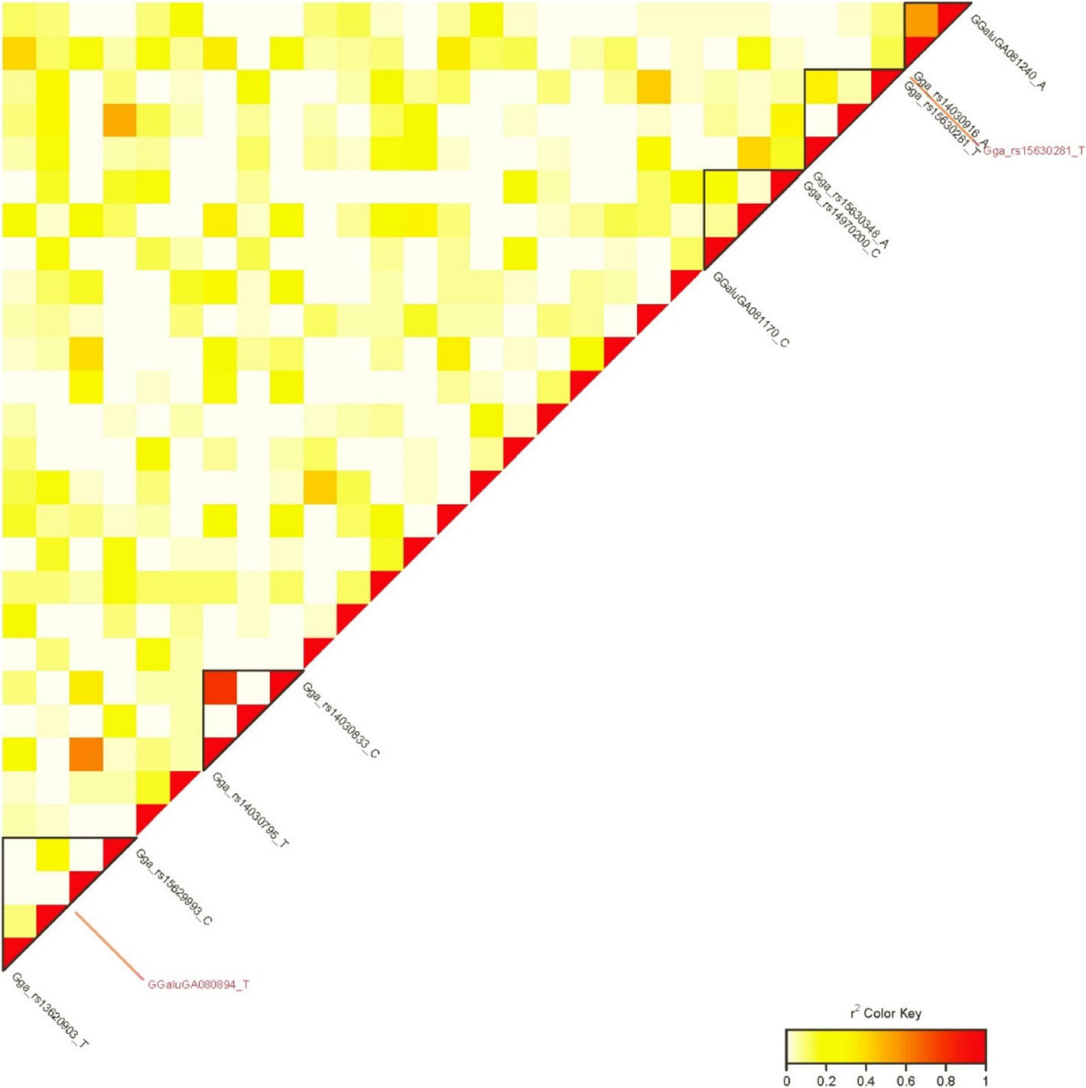
LD (r^2^) plot of markers on GGA12 associated with YEF

On GGA2, the significant SNP rs13730111 was located in the *ZNF704* (zinc finger protein 704) gene, and the *CA2* (carbonic anhydrase 2) gene neighboured the suggestive SNP rs316856766. On GGA12, LD analysis was performed for region 0.9 to 1.2 Mb (Fig. 3), and resulted in identifying the SNP rs315166929 in LD block of 27 kb and another SNP, rs15630281, between two haplotype blocks. The whole region contained a single gene, *RFT1* (RFT1 homolog), and the closest described gene, *PRKCD* (protein kinase C delta), situated 60 kb aside 25-kb LD block with high LD status (r^2^ > 0.75).

### Age at first egg

**Fig. 4:**
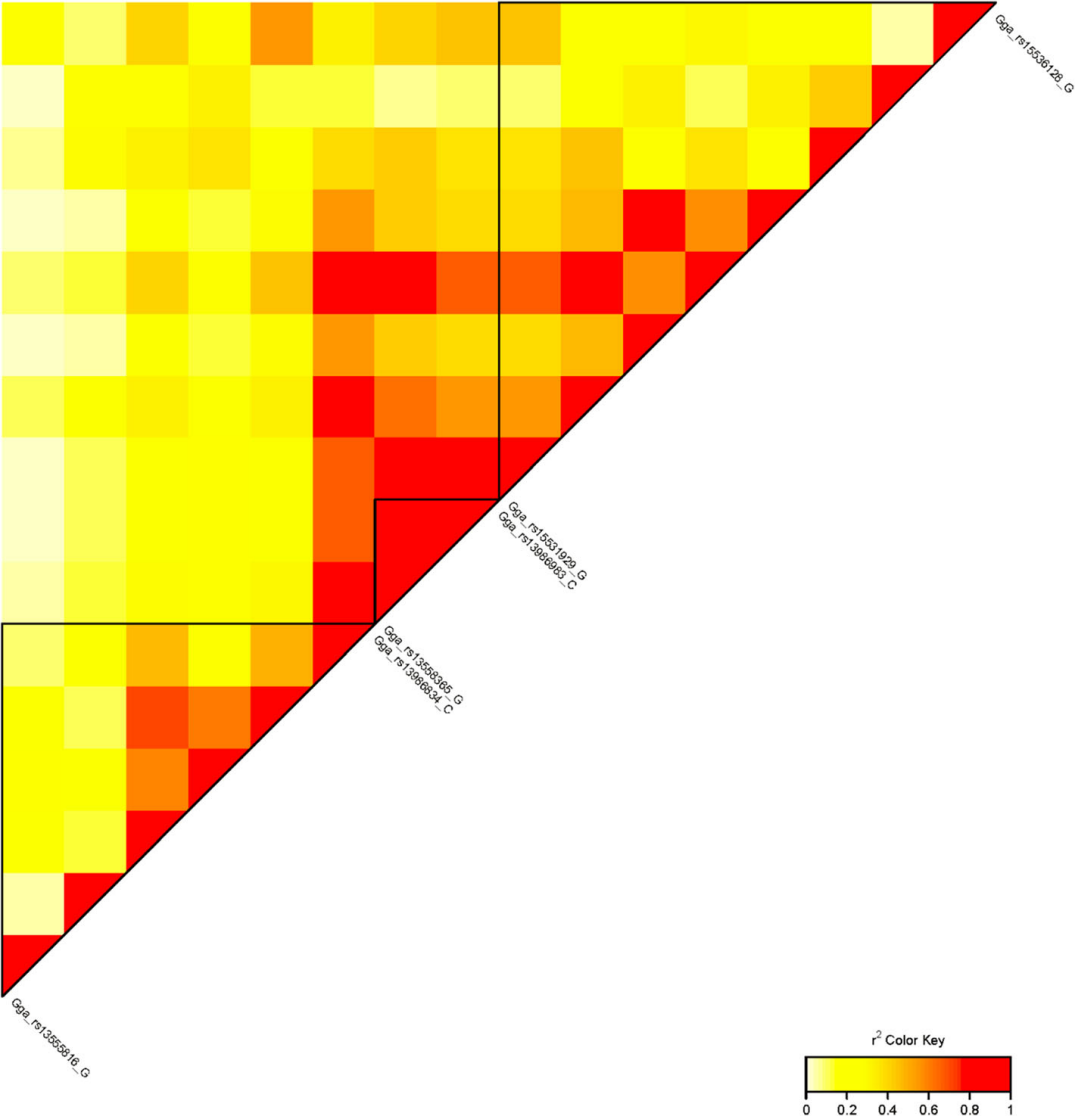
LD (r^2^) plot of markers on GGA1 associated with AFE

Only one marker (rs317931060) on GGA1 passed the suggestive line for AFE (Table 2), with *p*-value = 1.5e-5 and 13% (SE = 0.17) of phenotypic variance explained. Despite a low signal, we detected a quite remarkable peak below the suggestive marker line on GGA1 (*λ*_g_ = 0.995; Fig. 1). LD analysis was performed for 8-Mb region containing suggestive and peak SNPs with *p* ≤ 8.18e-4 (Fig. 4). Among three identified blocks, a suggestive SNP was located in a block with the lowest LD status as compared to others. The genes nearest to the suggestive 36-kb region were *DYNC2H1* (dynein cytoplasmic 2 heavy chain 1) and *PDGFD* (platelet derived growth factor D). We also noted a region with a high LD, containing the SNP rs13558365 and coinciding with the *DCUN1D5* (defective in cullin neddylation 1 domain containing 5) gene.

### Body weight

For BW, no SNPs were detected to be significantly associated with this trait. A single SNP, rs15619223, on GGA4 passed the suggestive line (*p* = 2.9e-5; *λ*_g_ = 1.019; Table 2 and Fig. 1), explaining 10.5% (SE = 0.14) of phenotypic variance. The marker was located within the *LCORL* (ligand dependent nuclear receptor corepressor like) gene.

### Egg weight

For EW, two suggestively associated SNPs were identified on GGA2 (Table 2), with the total explained phenotypic variance being 13% (SE = 0.14). These two SNPs, rs14201361 and rs14200974, were presented in the genes *KIAA1468* (lisH domain and HEAT repeat-containing protein) and *PHLPP1* (PH domain and leucine rich repeat protein phosphatase 1), respectively. Also, there was the *ZCCHC2* (zinc finger CCHC-type containing 2) gene between two SNPs in a region 68.3 to 68.5 Mb. One SNP on GGA4 was located on the suggestive line with *p*-value = 5.42e-5 and within the *TLL1* (tolloid-like protein 1 precursor) gene. The Manhattan and Q-Q plots for EW are presented in Fig. 1, with the observed *λ*_g_ being equal to 1.0106.

### Other traits

We did not detect any significant or suggestive associations for EM, EL and REFEW. The markers for EM and EL that had the greatest *p*-values were rs10724110 (*p* = 1e-4) on GGA2 and rs317565390 (*p* = 9.6e-5) on GGA8, respectively. Both markers are located in intergenic regions and have no links with any known genes. For REFEW, we identified the markers rs14196507 and rs13730111 on GGA2, rs315166929 on GGA12, and rs15763249 on GGA15 (*p* = 6.3*10–5 to 7.9*10–5). The SNP rs13730111 was also described in the given study as a significant marker for YEF. The second marker on GGA2 (rs14196507) is located in an intronic region of the *RREB1* (ras responsive element binding protein 1) gene. The markers on GGA12 and GGA15 are both located in intergenic regions with no link to known genes.

Also, we found genetic correlation between the pairs of traits EL and AFE, EL and EW, and EW and YEF that was respectively 0.48 ± 0.09, − 0.21 ± 0.05; and 0.82 ± 0.13 (*p* < 0.05).

## Discussion

### Day-old chick down colour

Typically, the down of RW chicks at day old is coloured in different shades of yellow. The first chicks with the white down were noticed during long-term selection for low temperature tolerance. Chicks with the white down were more tolerant to low temperatures and diseases, so breeders used colour as marker during the selection process [3]. Over the last decade, when research and selection process was ceased, white chicks still appeared in the progeny of the studied RW population. We found eight markers suggestively associated with DOCDC. The candidate genes located on GGA2 were *LRRCC1* encoding a centrosomal protein that maintains the structural integrity of the centrosome and plays a key role in mitotic spindle formation, *SLC7A13* encoding a protein mediating the transport L-aspartate and L-glutamate in a sodium-independent manner, and *ZFHX4* encoding a protein potentially playing a part in neural and muscle differentiation. Two extra genes, *LOC107052753* and *LOC101749450*, were uncategorised ncRNA loci. According to the Variant Effect Predictor (VEP) examination [37, 38], the SNPs rs15150566 and rs16116752 are intronic variants and modifiers of the genes *RALYL* and *ZFHX4*, respectively. The SNP rs14243963 is a synonymous variant meaning that has no impact on the *IMPA1* gene function. The *DYSF* gene on GGA4 encodes a skeletal muscle protein that is involved in muscle contraction and binds caveolin-3, a skeletal muscle membrane protein, which is important in the formation of caveolae. The three other found SNPs, rs16455118, rs317256404 and rs16209462, are located within introns. The *SPPL2B* gene on GGA28 encodes a member of the GXGD proteases that are transmembrane proteins with two conserved catalytic motifs localized within the membrane-spanning regions. This enzyme localizes to endosomes, lysosomes, and the plasma membrane. It cleaves the transmembrane domain, which triggers cytokine expression in the innate and adaptive immunity pathways. The function of the protein encoded by the *PLEKHM3* gene is characterized as metal ion binding and participation in skeletal muscle (myoblast) differentiation by acting as a scaffold protein for AKT1. None of the above genes may be directly related to pigmentation or DOCDC variants.

Thus, using a case-control GWAS approach for analysing phenotypes, we detected association with the genes responsible for immune system (*SPPL2B*) and muscle growth (*LLRCC1* and *ZFHX4*) in chickens. In the study by Psifidi et al. [39], several associations with immune resistance were also found on GGA2, but 20 Mb away from the loci detected in our study. Other associations were determined on GGA20, GGA11 and GGA13 [39] as well as GGA1, GGA5, GGA16 and GGA24 [36]. Better understanding of possible correlation between cold tolerance and the white DOCDC can be achieved by more accurate phenotypic data recording. Renewed programme of strong selection for cold tolerance will facilitate an increase in the number of phenotyped and genotyped individuals to find correlation between two traits by using common statistical approaches as the first step in future studies. At the same time, growing number of the genotyped animals subject to phenotypic recording will also improve sensitivity of the GWAS analysis. The observed genomic inflation factor (*λ*_g_) was close to 1, suggesting no population stratification. The Q-Q plot revealed a slight deviation from the distribution under the null hypothesis, which indicated a moderate association between SNPs and DOCDC.

### Yield of extraembryonic fluid

This trait is highly important in the process of producing embryo vaccines. Specific pathogen-free (SPF) eggs having no antibodies or pathogens are widely used for producing vaccines for animals and humans. Increasing volume of extraembryonic fluid results in a higher virus titre meaning an essential economical effect for biological preparation producers. The observed average YEF (9.5 ml; Table 3) means that three eggs are required to produce a single vaccine dose. Industry demand in establishing domestic poultry stocks producing SPF eggs for internal market inspired the current research targeting YEF in the RW breed. Trait variation in generation F_0_ was 17%, and selection of birds based on increasing of YEF level reduce it to 9.8% in F_3_. We did not find any previous GWASs on extraembryonic fluid, although some papers [10, 11] discussed selection in chickens based on the allanto-amniotic fluid volume, suggesting that because of a limited number of SPF egg producers there might be a restricted amount of information available.

**Table 3:** Basic statistics of recorded chicken performance data

Trait^a^	Observations	Min	Max	Mean	SD^b^	Heritability ± SE^c^
EW, g	146	43	61	50.1	3.4	0.53 ± 0.03
EM, g	146	5	11	9.2	0.9	N/A^d^
BW, g	146	1175	2360	1681.4	197.2	0.47 ± 0.01^e^
YEF, ml	146	5	15	9.5	1.7	0.17 ± 0.01
REFEW	146	0.1	0.3	0.2	0.03	N/A^d^
AFE, days	146	161	186	167.1	4.9	0.36 ± 0.01^e^
EL	146	76	194	142.5	20.8	0.21 ± 0.01

^a^Traits studied: *EW* egg weight, *EM* embryo mass, *BW* body weight, *YEF* yield of extraembryonic fluid, *REFEW* ratio of extraembryonic fluid to egg weight, *AFE* age at first egg, *EL* number of eggs

^b^*SD* standard deviation

^c^*SE* standard error

^d^*N/A* data not available

^e^Retrieved from data in Niknafs et al. [50]

The present study revealed two SNPs on GGA2 significantly and suggestively associated with YEF. Both markers are located in intronic regions. Close browsing of the region showed that significant marker was included in the *ZNF704* gene known to have expression in ovary and endometrium. The nearest to the suggestive SNP was the *CA2* gene responsible for encoding a protein that catalyses reversible hydration of carbon dioxide. Other suggestive regions were detected on GGA12, containing *RFT1* that is homologous to a yeast gene encoding an enzyme of the endoplasmic reticulum membrane in the pathway for the N-glycosylation of proteins, and the *PRKCD* gene encoding a protein from the kinase C family of serine- and threonine-specific protein kinases that is a positive regulator of the cell cycle progression and can positively or negatively regulate apoptosis. The *PRKCD* gene is expressed mainly in blood vessels and during their development, while one of the allantois functions is transport of oxygen, nutrients and excretion products, which is similar to the blood vessel system. The observed *λ*_g_ was lower than 1 and deviation of markers on the Q-Q plot was lower, suggesting a possible bias in GWAS.

In the study conducted by Psifidi et al. [39], several QTLs related to AFE and available in the Chicken QTLdb [40] were detected on GGA1, GGA2, GGA3, GGA4, GGA5, GGA7, GGA11, GGA13, GGA24 and GGAZ. A GWAS analysis of AFE presented by Yuan et al. [33] was based on White Leghorns and found suggestive associations on GGA16 and GGA23. Despite our expectations, we detected only one SNP suggestively associated with AFE on GGA1 and no significant associations. Within the SNP proximity we found three blocks with high LD structure. Two genes closest to an LD block that included the suggestive SNP were: *DYNC2H1* responsible for a protein involved in retrograde transport in the cilium and playing a role in intraflagellar transport, a process required for ciliary/flagellar assembly, and *PDGFD* encoding protein member of the platelet-derived growth factor family. Third gene, *DCUN1D5*, was found in an adjacent LD block and plays a part in neddilation of the *NEDD8* gene and SCF-complex (Skp, Cullin, F-box containing complex). Because *λ*_g_ was close to 1, no population stratification should be assumed. The Q-Q plot showed a slight deviation from the expected value, which indicated a moderate association between SNPs and AFE.

### Body weight

Previous studies showed a suggestive association of BW with a single SNP on GGA4 [41, 42]. The SNP detected in our investigation was in the *LCORL* gene, polymorphisms in which are linked with measures of skeletal frame size and adult height. Some GWASs [39, 41, 43] showed significant associations of BW with markers on GGA4. SNPs and genes described in previous reports were not concordant with our study, although they suggested a quite a narrow region between 60 and 80 Mb on GGA4 using the same 60 K SNP chip [39, 41, 43]. Limited number of genotyped birds, small population and direct phenotypic data can be possible explanation of a weak signal of SNPs in the given study. Despite the absence of significant associations, we presume the *LCORL* gene as a possible candidate responsible for birds growing and body weight improvement. The observed values on the Q-Q plot had negative deviation from the expected values, and at the same time *λ*_g_ was close to 1. In this particular case, the GWAS led to an association that can be treated as underestimated.

### Egg weight

Studies related to the association of EW with SNPs were done by Liao et al. [44] and Fan et al. [45]. Both studies focused on significant association with markers on GGA4, but several associations were also shown on GGA7, GGA3, GGA1 and GGA2. We found two suggestively associated SNPs that were 67.2 Mb away from rs14254270 on GGA2 published in the Fan et al. paper [45]. These were located within the *PHLPP1* gene encoding a protein, which promotes apoptosis by dephosphorylating and inactivating the serine/threonine kinase and conventional/novel protein kinase C (PKC) isoforms, and *KIAA1468* that participates in intracellular cholesterol transport. A single suggestive SNP was also detected on GGA4, and, as compared to previously shown results, the marker was situated 2 Mb aside from the published rs313911044 but close to *ZCCHC2*, with annotations related to this gene including nucleic acid binding and phosphatidylinositol binding. A high population stratification was shown by *λ*_g_ in EW, whereas the Q-Q plot reflected a low deviation of expected and observed markers.

### Other traits

Although there were no significant or suggestive SNP associations for EM, EL and REFEW, we additionally established genetic correlation for few pairs of phenotypic traits. The observed correlation can be used as a ground for multi-trait GWAS as described in Turley et al. [46]. In particular, EL may be analysed along with YEF as these traits had a high genetic correlation (*r* = 0.48 ± 0.09, *p* < 0.05). The traits EW and YEF were highly correlated (*r* = 0.82 ± 0.13, *p* < 0.05) and should be analysed together, as well. A similar correlation coefficient between EW and the absolute YEF value (*r* = 0.71 ± 0.03, *p* < 0.01) was previously reported for the Russian White breed, while that between EW and the relative YEF value was 0.31 ± 0.06 (*p* < 0.01) [47]. At the same time, the found negative correlation between EL and EW (*r* = − 0.26 ± 0.05, *p* < 0.05) comparable to that in other studies (e.g. -0.37 ± 0.06, [48]) should be taking into consideration because of an expected interest of farmers to get large number of eggs during laying period.

## Conclusions

Our study has represented the first GWAS analysis completed on Russian gene pool chickens. The significant and suggestive associations we found for YEF may serve as an important information for future allanto-amniotic fluid studies. The hypothetical association between the white DOCDC and cold tolerance in chicks will be investigated further and in more detail to provide a genetic source of adaptation in poultry. Studies on production traits provided essential information for future breed development and selection programme at the RRIFAGB Collective Use Centre ‘Genetic Collection of Rare and Endangered Chicken Breeds’ (CUC GCRECB). To assess potential of the found markers for marker-assisted selection (MAS), it would be desirable to perform a deeper analysis of suggestive regions using an expanded dataset and sequence information. The RW breed can be a valuable resource for local farmers and biotechnology sector, and the obtained data will further be used at the CUC GCRECB to select a breeding core for SPF eggs production.

In future studies, collection of additional phenotypic data and genotypes will be done for the RW breed, and MAS will be explored using SNPs that would explain a larger proportion of phenotypic variance. In addition, an extended GWAS can be done by using multi-trait analysis [46] for the correlated trait pairs identified in this study (EL–AFE, EL–EW and EW–YEF).

## Methods

### Samples and traits

Blood samples were collected from the wing vein of chickens by the standard venepuncture procedure. Animals were kept alive after blood collection and were not culled after the experiment. Genomic DNA was isolated from blood samples using a phenol/chloroform method [49]. DNA concentration ranged between 50 and 500 ng/μl. DNA quality and concentration corresponded to the requirements for the Illumina Infinium SNP genotyping platform.

The experimental chickens were a resource population of the RW breed maintained at the RRIFAGB CUC GCRECB. They were kept under the same conditions in individual cages and fed a commercial diet that contained 17% raw protein and 270 kcal energy per 100 g. In the current GWAS, 146 individuals representing progeny of 12 sires were tested. According to the pedigree, all animals descending from one sire were presented by half sibs (i.e. produced from different dams) and full sibs (from the same dam). The progeny data for each sire including number of half sibs, number of full sibs groups, mean number and standard deviation are given in Table 1. Eight animals with phenotypic records but unknown pedigree information were also included into the analysis.

The GWASs were performed using seven phenotypic traits: EW, YEF, REFEW, EM, EL, AFE, BW and DOCDC. For the EW, YEF and EM traits six sequentially laid eggs were determined per hen at age of 34 weeks. Liquid volume as a measure of YEF, and EM were found in 12.5-days embryos (see also Additional file 2). Total number of eggs (EL) was recorded during the egg laying period from first egg up to age of 52 weeks. BW of live hens was measured at age of 52 weeks. All data deviating by ±3 SD and more from a mean was excluded prior to analysis. DOCDC trait was recorded for genotyped animals by visual control on the first day after hatching. The case-control trait had two levels: white and yellow (Additional file 3: Figure S1).

Heritability estimates for EW, YEF and EL were computed by means of parent-offspring regression. Due to the lack of phenotypic records from parents for AFE and BW in the present study, their heritability values were derived from the data published by Niknafs et al. [50], while no heritability estimates were available for EM and REFEW. Descriptive statistics for quantitative traits used in association studies were calculated in RStudio [51]. Heritability and descriptive statistics are shown in Table 3.

### Genotyping

Genotyping was performed in the GeneSeek, Inc. laboratory (Lincoln, NE, USA) using Illumina Chicken 60KBeadChip. Quality control was carried out using the Plink1.9 programme [52]. SNPs were removed if they did not pass the following criteria: the call rate was less than 95%, minor allele frequency was lower than 0.01, missing rate per SNP was more than 20%, and Hardy-Weinberg equilibrium probability was less than 1e-4. SNPs on the sex chromosome GGAZ as well as linkage groups LGE64 and LGE22 treated as chromosomes were also excluded from the analysis. Missing SNPs were imputed using the Beagle 4.1 software [53]. The final SNP dataset was presented by 28 autosomes and embraced 35,390 SNPs. Marker information per chromosome is summarised in Additional file 1: Table S1. Extremely high SNP density on GGA16 resulted from lower number of SNPs used and smaller chromosome length.

### Whole genome association studies

A whole genome scan, with accounting of high relationship structure, was performed using a mixed model approach implemented in the Efficient Mixed-Model Association eXpedited (EMMAX) software [54]. SNP effect was computed using the following model: *Y* = *Xb* + *u* + *e*, where *Y* is a vector of phenotypes, *b* is a SNP effect, *X* is a design matrix of SNP genotypes, *u* is a vector of additive genetic effects assumed to be normally distributed with the mean equal to 0 and (co) variance ${\upsigma}_a^2$ G, with ${\upsigma}_a^2$ as the additive genetic variance and *G* as the genomic relationship matrix, and *e* is a vector of random residual effects. The genome-wide significance was assessed using the simple**M** method [41, 55] in R [56], for calculation of effective number of independent tests, *M*_*eff*_ . The significance and suggestive levels were set as 2.58*10^− 6^ (0.05/19,381) and 5.16*10^− 5^ (1.00/19,381), respectively. The Q-Q and Manhattan plots were derived from the GWAS results using the qqman package [57] within the R software. Genomic inflation factor (*λ*_g_) was computed based on *p*-values from the GWAS analysis by determining a ration between the median of the resulting chi-squared test statistics and the expected median of the chi-squared distribution in the R software. Estimation of phenotypic variance and heritability using the genomic-relatedness-based restricted maximum-likelihood (GREML) method in family data [58] was done by means of the GCTA software [59], threshold level for off-diagonals (make-bK parameter) being set at 0.5 that corresponds to close relationships among animals.

The post GWAS analysis included the LD determination of the chromosomal regions with significant SNPs that was performed using PLINK and Big-LD R-package [60]. Ensembl genome database including Gallus_gallus-5.0 genome browser and VEP [37, 38], NCBI databases [61] and GeneCards [62] were used for getting information about SNPs and relevant gene annotation.

## Additional files

Additional file 1: **Table S1.** Basic information for SNP markers per chromosome after quality control. (DOCX 29 kb)

Additional file 2: Description of the YEF and EM traits recording. (DOCX 13 kb)

Additional file 3: **Figure S1.** Day old chicks with the white (A) and yellow (B) down colour. (JPG 586 kb)

## Abbreviations

AFE: Age at first egg
bp: Base pair
BW: Body weight
CUC GCRECB: Collective Use Centre ‘Genetic Collection of Rare and Endangered Chicken Breeds
DOCDC: Day-old chick down colour
EL: Egg production
EM: Embryo mass
EW: Egg weight
GGA: Chicken (*Gallus gallus*) chromosome
GWAS: Genome-wide association study
kb: Kilobase
LD: Linkage disequilibrium
MAS: Marker-assisted selection
Mb: Megabase
ncRNA: Non-coding RNA
Q-Q: Quantile-quantile
QTL: Quantitative trait locus
REFEW: Ratio of extraembryonic fluid to egg weight
RRIFAGB: Russian Research Institute of Farm Animal Genetics and Breeding
RW: Russian White
SD: Standard deviation
SE: Standard error
SNP: Single nucleotide polymorphism
SPF: Specific pathogen-free
VEP: Variant Effect Predictor
YEF: Yield of extraembryonic fluid
