# Supplementary material for: Genome-wide association studies targeting the yield of extraembryonic fluid and production traits in Russian White chickens
Source: BMC Genomics. 2019 Apr 4;20:270. doi: 10.1186/s12864-019-5605-5 (PMC6449956; doi:10.1186/s12864-019-5605-5)
Supplement: Supplementary file 1 — Table S1. Basic information for SNP markers per chromosome after quality control. (DOCX 29 kb) [file 12864_2019_5605_MOESM1_ESM.docx]

**Table S1** Basic information for SNP markers per chromosome after quality control

| Chicken chromosome | Physical length (Mb)^a^ | No. of SNP markers | Density (kb/SNP) | Chicken chromosome | Physical length (Mb)^a^ | No. of SNP markers | Density (kb/SNP) |
| --- | --- | --- | --- | --- | --- | --- | --- |
| GGA1 | 200.9 | 6212 | 32.3 | GGA15 | 12.9 | 819 | 15.8 |
| GGA2 | 154.8 | 4613 | 33.6 | GGA16 | 0.4 | 3 | 116.9 |
| GGA3 | 113.6 | 3490 | 32.6 | GGA17 | 10.6 | 589 | 18.0 |
| GGA4 | 94.2 | 2800 | 33.6 | GGA18 | 10.9 | 678 | 16.1 |
| GGA5 | 62.2 | 1672 | 37.2 | GGA19 | 9.9 | 589 | 16.8 |
| GGA6 | 35.8 | 1396 | 25.7 | GGA20 | 13.9 | 1203 | 11.6 |
| GGA7 | 38.2 | 1384 | 27.6 | GGA21 | 6.8 | 503 | 13.6 |
| GGA8 | 30.5 | 1109 | 27.5 | GGA22 | 3.8 | 251 | 15.3 |
| GGA9 | 24.0 | 910 | 26.4 | GGA23 | 6.0 | 429 | 14.0 |
| GGA10 | 22.4 | 1073 | 20.9 | GGA24 | 6.4 | 516 | 12.3 |
| GGA11 | 21.9 | 1015 | 21.5 | GGA25 | 2.0 | 106 | 18.8 |
| GGA12 | 20.4 | 1127 | 18.1 | GGA26 | 5.0 | 438 | 11.5 |
| GGA13 | 18.3 | 928 | 19.7 | GGA27 | 4.8 | 326 | 14.8 |
| GGA14 | 15.8 | 789 | 20.0 | GGA28 | 4.5 | 411 | 10.9 |
| GGA15 | 12.9 | 819 | 15.8 | Total | 1009.6 | 35,390 | 28.5 |

^a^The physical length of the chromosome was based on the position of last marker on the genome build Gallus_gallus-5.0 [56]
