# Supplementary material for: Genome-wide association studies targeting the yield of extraembryonic fluid and production traits in Russian White chickens
Source: BMC Genomics. 2019 Apr 4;20:270. doi: 10.1186/s12864-019-5605-5 (PMC6449956; doi:10.1186/s12864-019-5605-5)
Supplement: Supplementary file 2 — Description of the YEF and EM traits recording. (DOCX 13 kb) [file 12864_2019_5605_MOESM2_ESM.docx]

**Description of YEF and EM traits recording**

Both traits were recorded using 6 sequentially laid eggs from one hen at age of 34 weeks. All eggs were incubated for 12.5 days days and placed after that for two additional days into a refrigerator. Eggs with an embryo were placed on tray keeping the larger end in the upward position. Eggshell covering the air cell was removed, and embryo and vitellus were pushed away for the allantois fluid aspiration using a pipet. As soon as all allantois fluid was removed, next steps were aspiration of the amniotic fluid using a clear pipet and extraction of the embryo. Amniotic fluid was measured using the volumetric flask. Embryo was placed on the analytical balance without vitellus to measure its mass.
