# Supplementary figures and images for: Genome-wide association studies targeting the yield of extraembryonic fluid and production traits in Russian White chickens
Source: BMC Genomics. 2019 Apr 4;20:270. doi: 10.1186/s12864-019-5605-5 (PMC6449956; doi:10.1186/s12864-019-5605-5)

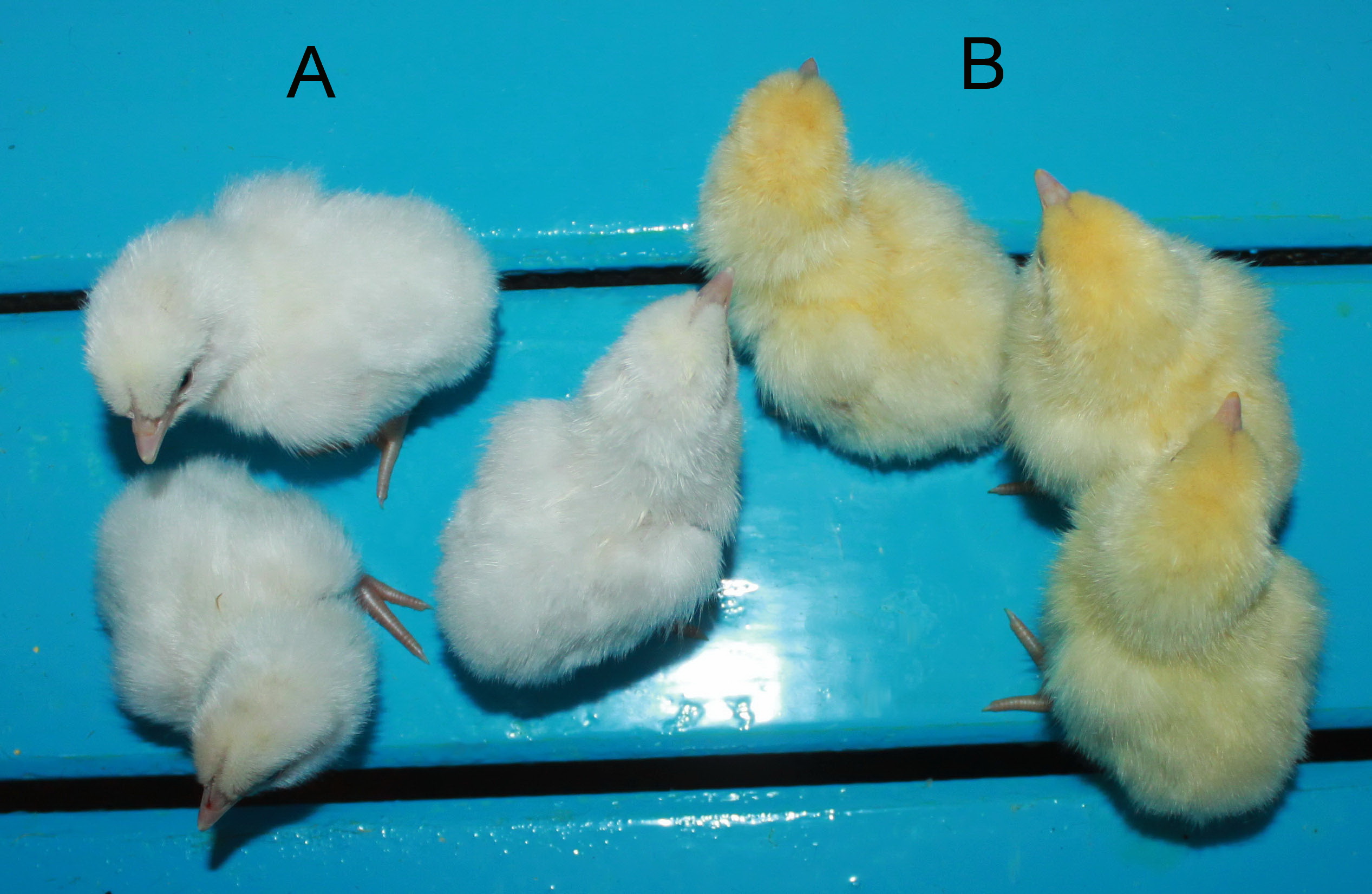

Supplement: Supplementary file 3 — Figure S1. Day old chicks with the white (A) and yellow (B) down colour. (JPG 586 kb) [file 12864_2019_5605_MOESM3_ESM.jpg]
